# Unbiased shRNA screening, using a combination of FACS and high-throughput sequencing, enables identification of novel modifiers of Polycomb silencing

**DOI:** 10.1038/s41598-018-30649-6

**Published:** 2018-08-14

**Authors:** Kenichi Nishioka, Hitomi Miyazaki, Hidenobu Soejima

**Affiliations:** 1 Division of Molecular Genetics and Epigenetics, Department of Biomolecular Sciences, Faculty of Medicine, Saga University, 5-1-1 Nabeshima, Saga City, Saga 849-8501 Japan; 2 Laboratory for Developmental Genetics, RIKEN IMS, 1-7-22 Suehiro-cho, Tsurumi-ku, Yokohama City, Kanagawa 230-0045 Japan

## Abstract

Polycomb silencing is an important and rapidly growing field that is relevant to a broad range of aspects of human health, including cancer and stem cell biology. To date, the regulatory mechanisms for the fine-tuning of Polycomb silencing remain unclear, but it is likely that there is a series of unidentified factors that functionally modify or balance the silencing. However, a practical gene screening strategy for identifying such factors has not yet been developed. The failure of screening strategies used thus far is probably due to the effect of the loss-of-function phenotypes of these factors on cell cycle progression. Here, by applying fluorescence-activated cell sorter (FACS) and high-throughput sequencing (HTS) technology in a large-scale lentivirus-mediated shRNA screening, we obtained a consecutive dataset from all shRNAs tested, which highlighted a substantial number of genes that may control Polycomb silencing. We consider that this unbiased strategy can readily be applied to a wide range of studies to uncover novel regulatory layers for expression of genes of interest.

## Introduction

Polycomb-group genes were originally identified using *Drosophila* genetics, and have been shown to regulate axial body pattern formation by silencing numerous homeotic genes^1,2^. In mammals, these repressor proteins are thought to be necessary for regulation of the pluripotency and self-renewal of various tissue stem cells, or even for their differentiation during development, which they would achieve by controlling cell-fate determination^3^. Mutations in Polycomb-group genes, as well as histone genes encoding their targets, have been found in numerous human cancers^2–4^, resulting in the rapid expansion of research into this important field, which is relevant to a broad range of aspects of human health.

Polycomb-group proteins are classified into two major classes of biochemically distinct multiprotein complexes: Polycomb repressive complex 1 (PRC1) and 2 (PRC2)^1^, which exhibit distinct enzymatic activities that involve using histones as substrates. The Ring1a/b subunits of PRC1 are E3 ubiquitin ligase to act on histone H2AK119, while the Ezh1/2 subunits of PRC2 mediate the methylation of histone H3K27. These complexes have recently been further subdivided into a series of complexes based on the presence of unique accessory proteins: PRC1.1-1.6 and PRC2.1/2.2^5–7^.

A principal repression mechanism by which Polycomb silencing works is PRC1/2-mediated chromatin compaction^8–10^, whereby transcription factors and chromatin remodelling factors are excluded. Therefore, it is expected that each subclass of complexes may govern a dif ferent repression level, although the precise mechanism of repression remains unclear. However, recent evidence has shown that the PRC2-mediated histone H3K27me3 plays the most important role in stabilising repression^11,12^.

In addition to repression, PRC1/2 play a role in gene activation, and there is solid evidence that Polycomb-group proteins can act directly in this capacity^13–16^. Thus, transcriptional regulation by PRC1/2 is complex.

Based on the complexity of Polycomb silencing, we hypothesised that this silencing is modified or balanced by a series of unidentified factors. Identification of these factors may help us to understand the underlying mechanisms for the fine-tuning of Polycomb silencing. A previous screening strategy to identify these factors relied on colony formation upon de-repression of the *Gata6*-promoter-driven neomycin-resistance gene^17^. However, a loss-of-function phenotype of Polycomb silencing modifiers may negatively affect cell cycle progression and/or cell viability, possibly resulting in important novel factors or even Polycomb-group genes themselves being missed. While an array-based strategy can address this weakness, it is expensive and requires specialised equipment, and would provide a limited number of testable genes^18,19^. Thus, a practical gene screening strategy for the identification of Polycomb silencing modifiers is yet to be developed.

Here, we applied fluorescence-activated cell sorter (FACS) and high-throughput sequencing (HTS) technology to a large-scale lentiviral shRNA library screening, and the resulting consecutive dataset of tested shRNAs enabled us to overcome the problems described above. The use of an shRNA library enabled us to perform an inexpensive, large-scale screening. Importantly, the simultaneous sampling of the input and desired fractions using FACS (a common piece of equipment in medical institutes) minimised the artificial effects resulting from differences in cell growth. Using this screening strategy, we identified a number of Polycomb-group genes as controls. In particular, we identified important PRC2 encoding genes, as well as a number of genes that are reportedly closely linked to Polycomb silencing. We also briefly discuss our recent publication regarding Mbf1^20^, as well as characterisations of two potential Polycomb silencing modifiers, Prdm5 and Setd5, using published mouse embryonic stem cell (ESC) datasets. We propose that based on the principle of this method, by changing the reporter and cell type, this unbiased screening strategy can readily be applied in a wide variety of contexts to uncover novel regulatory layers of the expression of genes of interest.

## Results and Discussion

### Mouse F9 cell selection

Mouse F9 embryonic carcinoma stem cells are a well-characterised stem cell line widely used to mimic differentiation into parietal endoderm cells, which is a part of initial step in the differentiation process during early mouse development^21^. *In vitro*, parietal endoderm-like cells are induced by retinoic acid (RA) and dibutyryl cAMP (db-cAMP). Importantly, F9 cells grow rapidly without feeder cells and have good viability and plating efficiency. Furthermore, differentiated F9 cells do not exhibit complete cell cycle arrest. All of these are advantageous for cell-based gene screening.

### Reporter gene under control of Polycomb silencing

**Figure 1:**
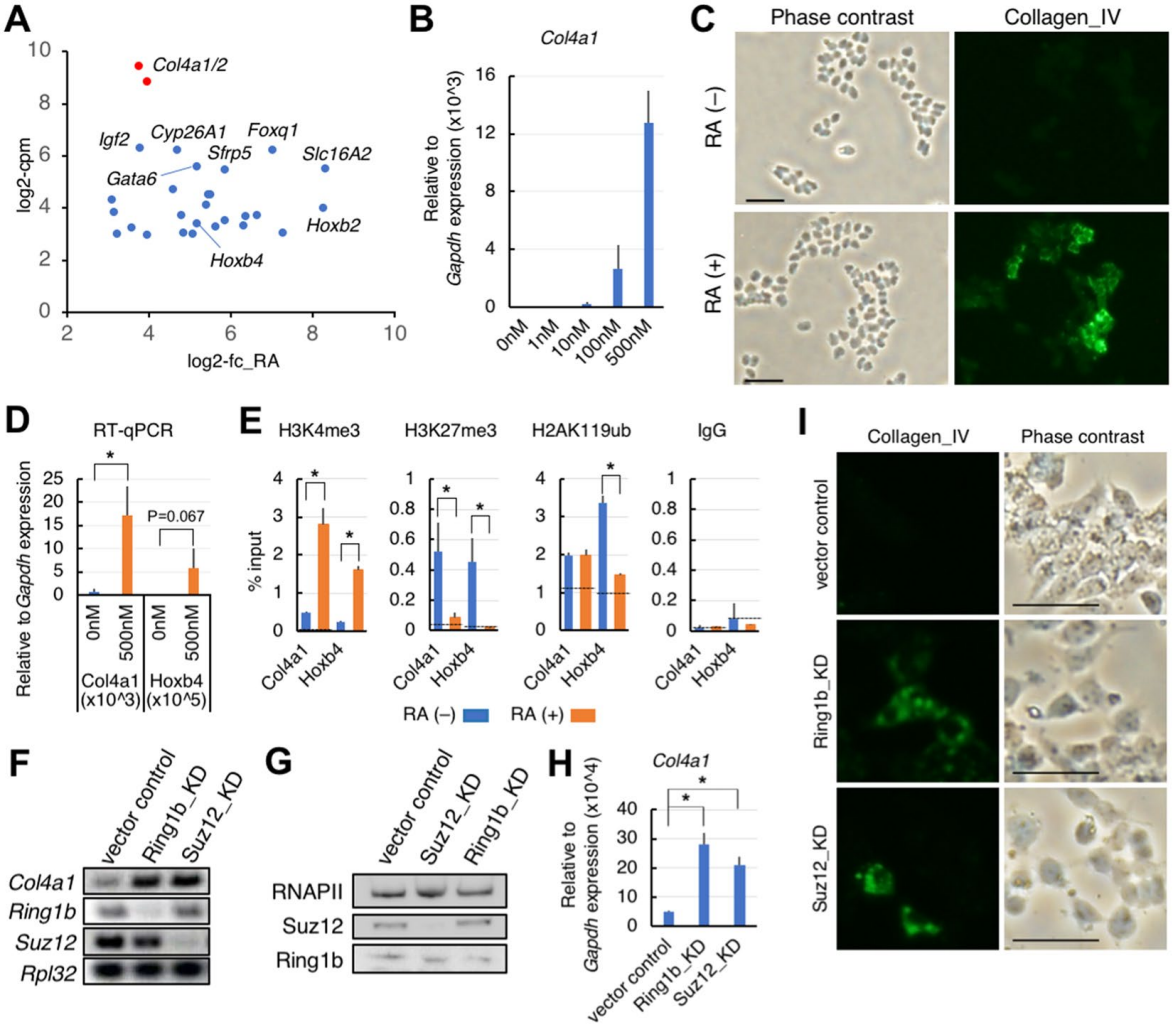
Characterisation of mouse F9 cells for screening purposes. (**A**) A scatter plot of candidate reporter genes in F9 cells. Upon RA treatment, 28 Polycomb-target genes were strongly upregulated. X-axis, log2-fold change (log2-fc) in gene expression after RA treatment; Y-axis, mean log2- count per million (log2-cpm) representing relative expression levels. (**B**) RT-qPCR of *Col4a1* mRNA expression in differentiating F9 cells. RA was titrated in the presence of db-cAMP. Data presented as mean ± s.d. relative to *Gapdh* mRNA expression. (**C**) Immunofluorescence analysis of type IV collagen expression before (−) or after (+) RA-induced differentiation. Bars, 50 µm. (**D**) RT-qPCR f *Col4a1* and *Hoxb4* mRNA expression at the indicated RA concentration. Data presented as mean ± s.d. relative to *Gapdh* mRNA expression. *P < 0.05. (**E**) ChIP-qPCR analysis of histone H3K27me3, H3K4me3 or H2AK119ub levels in chromatin containing the indicated gene promoter regions (around +300 bases from the transcription start site) in the presence or absence of RA. Data presented as mean ± s.d. relative to input. ChIP signals from the promoter region of *Il2ra* (control region) were indicated by interrupted lines. *P < 0.05. (**F**) Conventional RT-PCR of indicated mRNA expression in either *Suz12* or *Ring1b* knockdown (KD) cells. (**G**) Western blot analysis of KD cells using the indicated antibodies. (**H**) RT-qPCR of *Col4a1* mRNA expression in the indicated KD cells. Data presented as mean ± s.d. relative to *Gapdh* mRNA expression. *P < 0.05. (**I**) Immunofluorescence analysis of type IV collagen expression in the indicated KD cells. Bars, 50 µm.

From the candidate reporter genes for identifying Polycomb silencing modifiers, we chose type IV collagen, because this protein is highly induced during the differentiation of F9 cells^22^. Using recent high-throughput datasets^23,24^, we performed *in silico* selection of candidate reporter genes (Supplementary Fig. S1A) and then extracted 28 RA-inducible Polycomb target genes. Many of the extracted genes (23 genes) are members of the PRC module in ESCs^25^. Based on expression levels of the top-ranked genes, we chose *Col4a1/a2*, encoding the alpha-1 or alpha-2 chain of type IV collagen, for the reporter gene in our shRNA screening (Figs 1A and S1B). We then analyzed *Col4a1*(*/2*) expression by our hands. Reverse transcription-quantitative polymerase chain reaction (RT-qPCR) analyses of *Col4a1* mRNA expression showed a wide range of expression levels when treated with serial concentrations of RA (Fig. 1B). Type IV collagen was visualised by immunofluorescence microscopy as an intracellular structural protein produced in response to RA treatment (Fig. 1C). Chromatin-immunoprecipitation (ChIP) analyses of a promoter-proximal coding region of *Col4a1* revealed that chromatin was bivalently modified by both histone H3K4me3 and histone H3K27me3 (Fig. 1E), indicating that *Col4a1* was a Polycomb-regulated gene, similar to *Hoxb4*. Importantly, upon RA treatment, expression of *Col4a1* and *Hoxb4* mRNAs was negatively correlated with the level of histone H3K27me3 (Fig. 1D,E). This result suggested that *Col4a1* mRNA expression was regulated by alleviating Polycomb silencing. The *Col4a1/a2* promoter region is also bivalently modified in ESCs (Supplementary Fig. S1C), and these genes were reportedly assigned to the PRC module^25^.

We next tested whether a knockdown of Polycomb-group gene products could induce expression of *Col4a1*. RT-PCR analyses of each knockdown showed upregulation of *Col4a1* mRNA (Fig. 1F–H). Moreover, immunofluorescence microscopy analysis revealed induction of type IV collagen by Ring1b- or Suz12-knockdown (Fig. 1I). These results provided promising evidence that type IV collagen was a suitable reporter for detecting a loss-of-function phenotype of Polycomb silencing.

### The principle of target cell isolation, and some methodological recommendations

Having established that type IV collagen was a suitable reporter for our shRNA screening, we carefully sought a suitable screening strategy, since a previously reported gene screening using a Polycomb-target gene as the reporter failed to enrich most Polycomb-group genes^17^. Given that the major functions of Polycomb silencing are cell lineage commitment and cell cycle control, a loss-of-function phenotype of Polycomb silencing modifiers may inhibit cell cycle progression, and this may adversely affect a colony-based screening strategy by thresholding surviving cells, and which usually lacks a reference of non-selected cells, giving a substantial number of false negatives.

**Figure 2:**
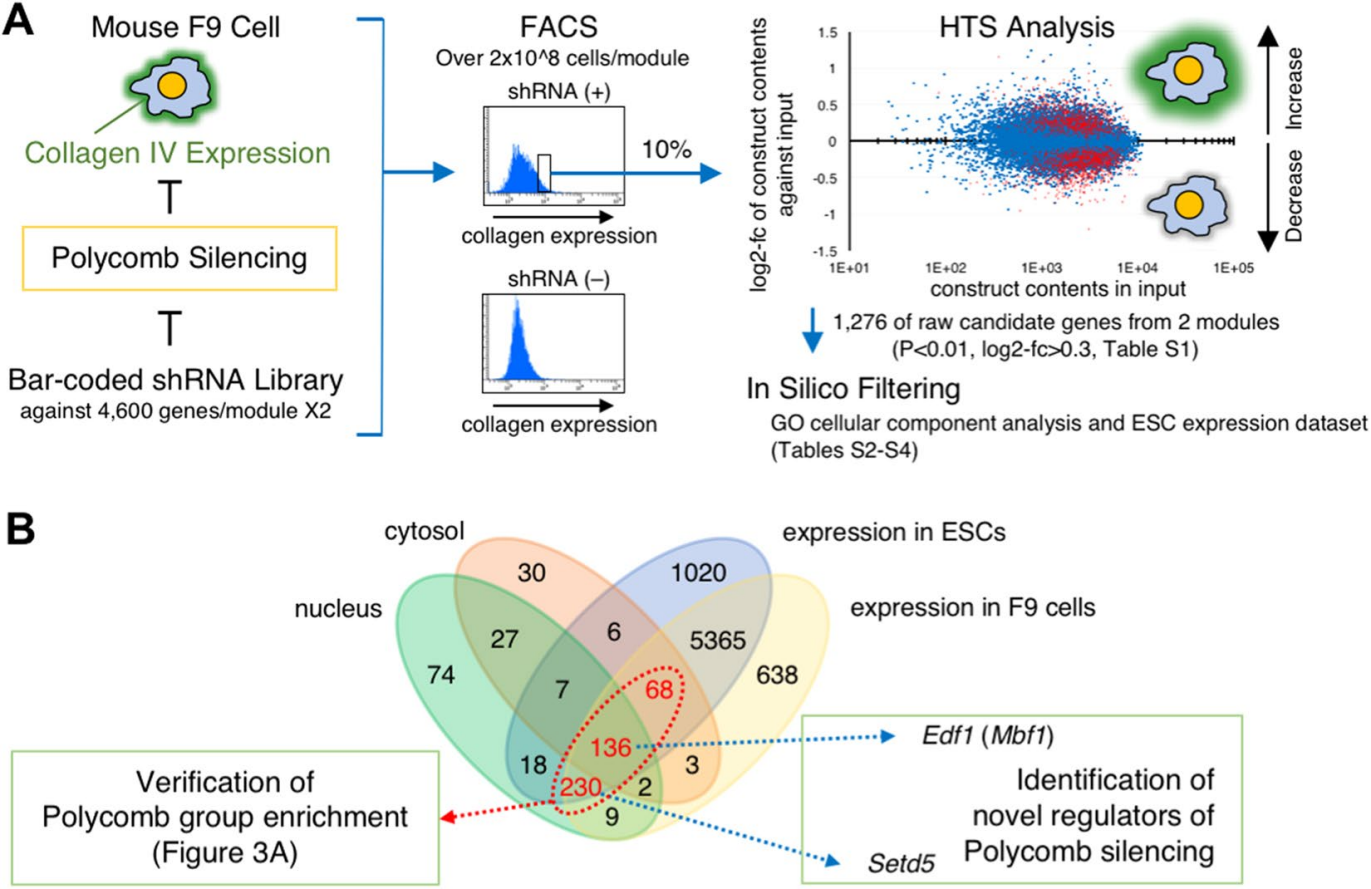
Outline of our screening strategy. (**A**) Lentiviral transduced F9 cells (>2 × 10^8^ cells) were subjected to FACS, and then the input fraction (2 × 10^7^ cells) and the Allexa488-high-intensity fraction (2 × 10^7^ cells) were pooled in each replicate. Genomic DNA was processed for HTS library generation, and 2 × 10^7^ reads for each fraction were sequenced. Scatter plot representing each shRNA construct in the high-intensity fraction against input. X-axis, normalised representation of input; Y-axis, log2-fc of normalised high-intensity fraction against input. Red spots represent shRNA constructs that changed significantly relative to the input (P < 0.01), of which the upper group was used in this study (Supplementary Table S1). The resulting 1,276 raw candidate genes were subjected to *in silico* filtering, with 434 final candidate genes extracted (Supplementary Tables S2–S4). (**B**) A Venn diagram representing the relationships among the extracted candidate genes and their expression in F9 cells and ESCs. The 434 final candidate genes (shown in red) were subjected to further verification analysis for Polycomb-group enrichment (see Fig. 3A), with *Edf1* (*Mbf1*) and *Setd5* selected for further characterisation. Data are summarised in Supplementary Table S3.

To overcome this source of selection bias, we avoided colony formation and instead employed FACS, sampling enough cell volumes for both the reference input (non-selected) and selected fractions simultaneously; thus we intended to recover any false negatives that would have been produced as suggested above by extracting all the information for the shRNAs tested. Figure 2A outlines our screening strategy. The pooled shRNA library used was composed of two modules, each containing 27,500 shRNA constructs against approximately 4,600 mRNAs. A quality check by the manufacturer revealed that representations of the shRNA constructs in the modules varied, but there was less than a 100-fold difference among them. In this study, we established 6–7 × 10^6^ independent cell clones (>200×) for each module. Nevertheless, considering possible cell growth bias from library transduction, we planned to obtain 2 × 10^7^ cells for each pooled fraction from analysis by FACS (the input and the Alexa488-high-intensity fractions) so that we could analyse all the shRNA constructs in the library. Although this number may seem large, we nevertheless had several shRNA constructs for which the read counts were in single digits. Accordingly, we sorted more than 2 × 10^8^ cells (>10 × the number in each fraction) as the total input for each replicate. This number of cells for the total input, and 10% of this number for pooled fractions, would therefore be the minimum required to obtain consecutive enrichment scores for all the tested shRNA constructs.

Regarding the final preparation of cells for FACS, we observed that cell density affected the expression level of type IV collagen: high-cell-density cultures continued to have low expression levels (Supplementary Fig. S2), conferring false negatives. Therefore, the cell density was maintained below 2.5 × 10^4^ cells/cm^2^ at the final splitting in 100 mm plates. We chose methanol-PBS (9:1, v/v) as the fixative, because it permeabilises cells, terminates the TagRFP marker protein signal derived from the integrated shRNA construct, and preserves cell morphology. To avoid cell loss during staining procedures, all procedures were performed in the presence of 10% serum. This supplement was very important, because using siliconised tubes was not effective for avoiding such cell loss. However, if cells are not permeabilised, serum might not be required. Finally, other dyes could be used for the label attached to the secondary antibody, for example any far-red dyes could be used, if required, although they might become photobleached over several days of cell sorting.

### Primary selection and filtering of identified candidates

**Figure 3:**
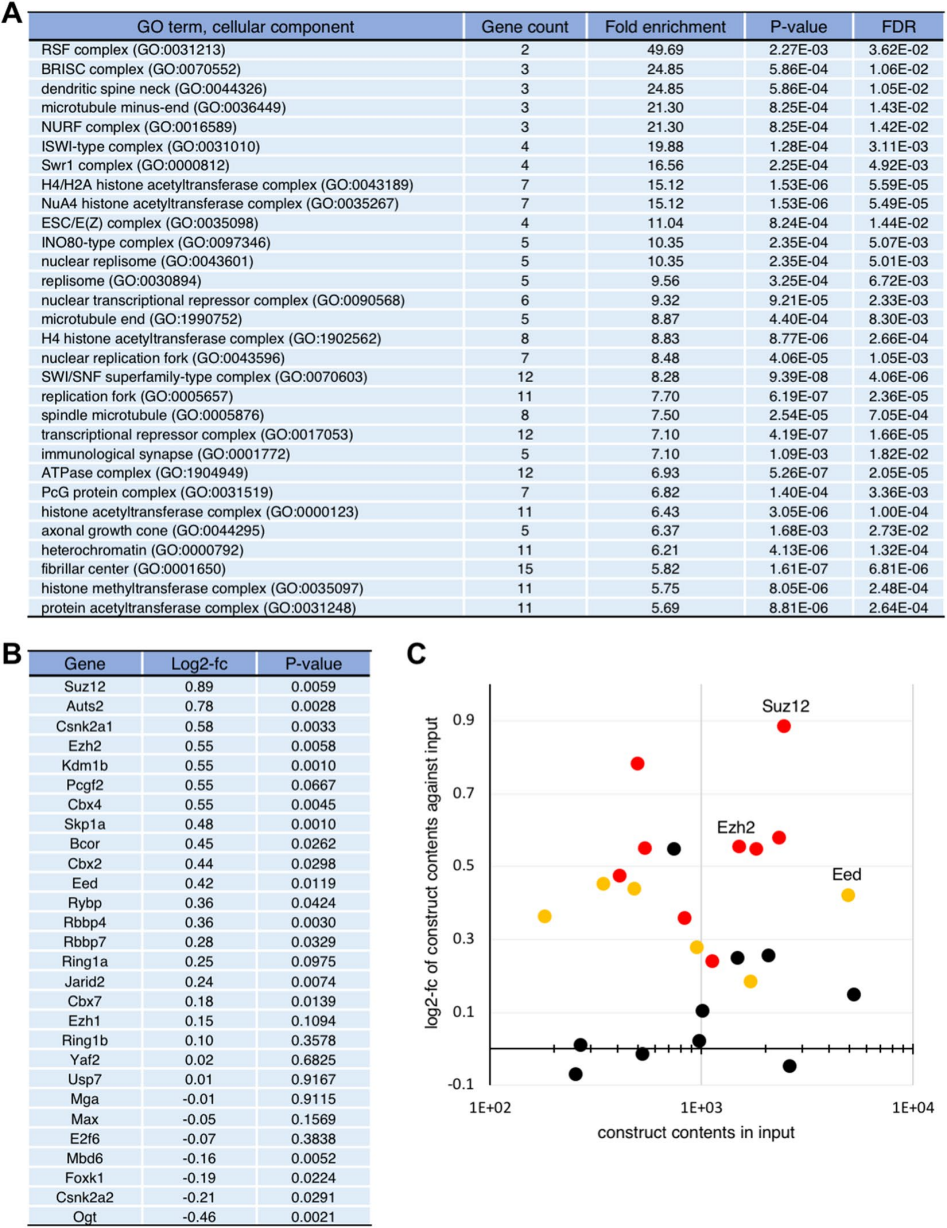
Evaluation and verification of our screening strategy. (**A**) The 434 final candidate genes (Supplementary Table S3) were analysed using the PANTHER classification system with regard to cellular component enrichment. The top 30 terms ranked according to fold enrichment are shown. (**B**) Screening results of the representative shRNA constructs for each Polycomb group gene or its related gene are listed^2^. (**C**) Scatter plot of 25 of a part of genes listed in (B), with significantly enriched shRNA constructs represented by coloured spots (orange, P < 0.05; red, P < 0.01). PRC2 core components are labelled.

By using HTS analyses, we obtained information regarding read counts for all shRNA constructs and their target mRNAs in the pooled fractions: the input fraction and the Alexa488-high-intensity fraction. Log2-fold change (log2-fc) and P-values for differences between the input and the Alexa488-high-intensity fractions were calculated for each shRNA construct from three biological replicates. We applied loose criteria to our consecutive dataset for primary selection (P < 0.01 and log2-fc > 0.3), extracting 1,276 candidate genes from the tested modules (Supplementary Table S1). These cut-offs for selection were supported by later validation, as shown in Fig. 3. The primary candidate genes were filtered using gene ontology cellular component analysis, the F9 cell-expression dataset, and the ESC-expression dataset, resulting in 434 final candidate genes (Fig. 2B and Supplementary Tables S2–S4). The purpose of using in silico selections in this step was to isolate genes that directly modify either the expression processes or the functions of Polycomb-group proteins, and to generalise our results to common stem cells.

### Verification of results

Using gene ontology cellular component analysis, we determined whether Polycomb-group genes were enriched in the 434 final candidate genes. Intriguingly, we found significant enrichment of a series of genes encoding chromatin modifiers, chromatin remodelling complexes, and replication complexes (Fig. 3A). These genes may have a function that is closely related to Polycomb silencing, either directly or indirectly. Most importantly, we found “ESC/E(Z) complex” and “PcG protein complex” as significantly enriched terms.

Pathway analysis of these 434 genes highlighted several signaling pathways including “p53 Pathway” and “Ras Pathway” (Supplementary Table S5). Although we did not perform any further validation regarding these pathways, these results should be intriguing to researchers in the relevant fields.

We next verified our results regarding Polycomb-group and related genes encoding components of the PRCs^2^. Figure 3B shows the representative shRNA constructs for each gene tested. We found more than half of the tested genes demonstrated significant enrichment in the high-intensity fraction relative to input (P < 0.05). Importantly, shRNA constructs against *Suz12* and *Ezh2* mRNAs, in particular, which encode core components of the PRC2, were relatively enriched. By plotting this result, we found that log2-fc > 0.3 was an appropriate cut-off (Fig. 3C), and then applied it in general. Thus, we concluded that our unbiased screening strategy was fit for the purpose of identifying Polycomb silencing modifiers.

It is also important to select an appropriate cell type. For example, human AUTS2 and CSNK2A1 in the context of human PRC1.5 have been shown to play a role in gene activation in the central nervous system^14^. In this study, we identified these as positive modifiers of Polycomb silencing, which may be inconsistent with the previous report. Since F9 cells are committed to differentiating into parietal endoderm-like cells, differentiation toward neuronal cells should be suppressed under normal conditions. In addition, Mga, Max and E2f6, all of which are components of PRC1.6, were rather underrepresented in the high-intensity fraction (Fig. 3B). Moreover, our results using F9 cells were clearly distinct from those of Cooper and Brockdorff^17^, who used ESCs, despite the screenings being conceptually similar, in that they were both based on the use of representative Polycomb-target genes as reporters (Figs 1A and S1B). This previous screening was a sole example with the intention to identify Polycomb silencing modifiers in mammal. Genes encoding PRC2 subunits were found only in our candidates, and no common Polycomb-related gene was found between our candidates and theirs (Supplementary Fig. S3). These results suggest that screening results will differ in a context-dependent manner, which we attribute to the type of cells used.

We failed to see enrichment of the *Ring1b* shRNA construct in the high-intensity fraction (Fig. 3B). However, when we designed another shRNA construct targeting a different region of *Ring1b* mRNA, we clearly observed upregulation of *Col4a1* upon knockdown (Fig. 1H and I). These results indicate that the library we used is still under development and should be updated. This limitation should be considered carefully.

### Identification of novel Polycomb silencing modifiers

Having shown that our screening was successful, we further researched several candidate genes. Rsf1 was recently reported to be a histone H2AK119ub1-binding protein, and 82% of H2AK119ub1-enriched genes are co-localised with Rsf1^26^. Rbm15 has been identified as a factor necessary for X-chromosome inactivation^27^, which is a representative context regulated by Polycomb silencing. Human CDYL has an intimate functional relationship with Polycomb silencing^28^. Moreover, human NIPP1 (also called PPP1R8) was shown to be a PRC2-dependent transcriptional repressor that maintains EZH2 phosphorylation^29^. These reports collectively support the reliability of this screening method.

**Figure 4:**
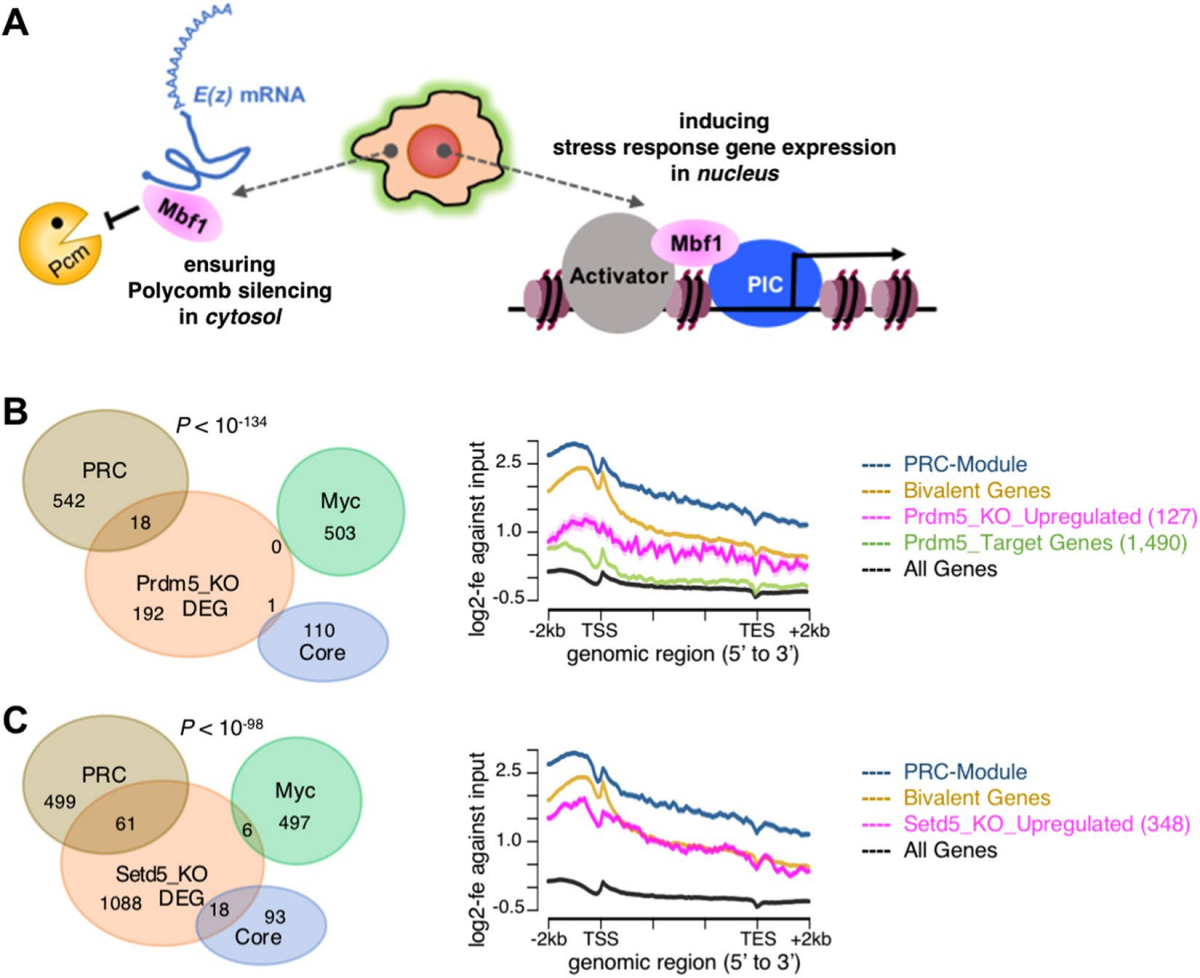
Examples of newly identified genes. (A) A graphical summary of a novel function of Mbf1, the *Drosophila* counterpart of mammalian Edf1. Mbf1 is a known nuclear coactivator promoting an interaction between an activator and a transcriptional preinitiation complex under stress conditions. We recently reported that cytosolic Mbf1 protects *E*(*z*) mRNA from Pacman attack under non-stress conditions, thereby ensuring robust Polycomb silencing^20^. (**B** and **C**) HTS analyses of (B) *Prdm5* and (C) *Setd5*. Left panel, Venn diagrams analysing the impact of the differentially expressed genes (DEG) in each knockout (KO) in the ESC modules. DEG, |log2-fc| > 1 and P < 0.01. Statistical analysis of the relationship between the PRC module against either the Myc module or the Core module was done by the Chi-square test. *Prdm5*, *P* < 10^−134^; *Setd5*, *P* < 10^−98^. Right panel, metagene analyses of the histone H3K27me3 level in the indicated genomic region by plotting log2-fold enrichment (log2-fe) against input data. PRC-Module, Kim *et al*.^25^; Bivalent Genes, Mikkelsen *et al*.^42^.

Furthermore, the present screening identified Edf1 as a novel candidate Polycomb silencing modifier (Figs 2B and 4A). Previously, we fully characterised a functional relationship between Polycomb silencing and Mbf1, the *Drosophila* counterpart of mammalian Edf1^20^. Mbf1 is a dual subcellular localisation protein and is mainly localised in the cytosol under non-stress conditions. We have shown that cytosolic Mbf1 protects *E*(*z*) mRNA from Pacman attack, thereby ensuring robust Polycomb silencing.

Here we present two additional identified genes, *Prdm5* and *Setd5*, which were expressed in ESCs (Supplementary Table S4). Functional characterisations of these gene products have been produced using knockout ESCs^30,31^, but their relationship to Polycomb silencing remains to be elucidated. Using publically available HTS datasets, we examined whether the upregulated gene sets in each knockout context were under the control of Polycomb silencing. The results regarding *Prdm5* are shown in Fig. 4B. The effect of differentially regulated genes on the ESC modules was strongly biased towards the PRC module (Fig. 4B, Venn-diagram). The histone H3K27me3 level in either the upregulated genes or the Prdm5-target genes were substantially enriched compared to that in the average of all genes (Fig. 4B, metagene analysis). Although ChIP-seq data were not available, RNA-seq analysis of *Setd5*-knockout ESCs showed largely similar results to those of *Prdm5*-knockout ESCs, with more prominent histone H3K27me3 enrichment (Fig. 4C). These data provided promising evidence that both Prdm5 and Setd5 are potential modifiers of Polycomb silencing, although further characterisations should be done.

### Concluding remarks

In summary, to identify novel Polycomb silencing modifiers, we performed a large-scale shRNA screening using FACS and HTS technology to obtain a consecutive and unbiased dataset. By using this strategy with different cell types and reporters, other novel candidates that modify Polycomb silencing may also be identified. Furthermore, the strategy presented here should also be applicable to a wide variety of screening studies to uncover a novel regulatory layer of gene expression.

## Methods

### Cell culture

Mouse F9 embryonic carcinoma cells were obtained from RIKEN Bioresource Center. The cells were maintained in DMEM (Invitrogen) containing 1X GlutaMAX-I (Gibco), 1X MEM NEAA (Gibco), 0.1 mM 2-Mercaptoethanol (Gibco), 50 units/ml penicillin (Gibco), 50 µg/ml streptomycin (Gibco), 10% fetal bovine serum (FBS). F9 cells were differentiated into parietal endoderm-like cells by the addition of all-trans retinoic acid (RA; Sigma) for one or two days in the presence of 1 mM dibutyryl cyclic AMP (db-cAMP; Sigma).

### Immunofluorescence microscopy

F9 cells were fixed in methanol-acetone (1:1, v/v), treated with blocking buffer (PBST with 1% skim milk), and then probed with 10 µg/ml of anti-collagen IV antibody (Abcam, ab19808) in the blocking buffer overnight. After washing the cells with PBST, the type IV collagen expression was visualised by goat anti-rabbit IgG antibody conjugated with Alexa488 (Molecular Probes)

### Chromatin-immunoprecipitation (ChIP)-quantitative polymerase chain reaction (qPCR)

ChIP was performed as previously described^32^, using anti-histone H3K4me3 (Millipore, 07–473), H3K27me3 (Millipore, 07–449), H2AK119ub (CST, D27C4), or control rabbit IgG. Immunoprecipitated DNA was purified using a PCR Purification Kit (Qiagen) and quantified by qPCR. The PCR primer pairs used are listed in Supplementary Table S8. Each result and error bar graphed represents the percentage input of the mean ChIP signal in each region and standard deviation (s.d.) calculated from three biological replicates. The ChIP signal in a promoter region of *Il2ra* was used as a background control. Statistical significance was tested using the Student’s *t*-test.

### RT-PCR

Total RNA was extracted from cells using Isogen II (Nippongene) and cDNA was synthesised using a Transcriptor First Strand cDNA Synthesis Kit (Roche) according to the manufacturer’s instructions. cDNAs were subjected to conventional PCR or qPCR with SYBR green dye on a LightCycler 480 machine (Roche) using the primer pairs listed in Supplementary Table S8. Each qPCR result and error bar graphed represents the fold enrichment of the normalised mean qPCR signal of the target mRNA in each sample against that in the empty vector control sample ± s.d., calculated from three biological replicates. Data normalisation was performed using the *Gapdh* mRNA level in each sample. Statistical significance was tested using the Student’s *t*-test. The raw gel data from the conventional PCR are shown in Supplementary Fig. S4A.

### Western blot

Nuclear extracts (35 µg each from control and knockdown cells) were prepared and loaded onto 5–20% SDS-PAGE gels. Resolved proteins were electronically blotted onto PVDF membranes (GE healthcare). After blocking the membrane strips with 3% skim milk, they were probed with either anti-RNA polymerase II antibody (Covance, 8WG16), anti-Ring1b antibody (CST, D22F2), or anti-Suz12 antibody (CST, D39F6), and signals were visualised using the enhanced chemiluminescence system.

### Lentiviral transduction and cell culture

We used the Decipher pooled lentiviral shRNA libraries (DECIPHER, Cellecta), which carry a specific barcode for each shRNA construct, the TagRFP marker gene encoding a monomer red-fluorescent protein, and the puromycin-resistance gene. The libraries were composed of two modules: Module 1 (DMPAC-M1-P, signalling pathways, 27,500 shRNAs against 4,625 mRNAs) and Module 2 (DMDAC-M2-P, disease-associated, 27,500 shRNAs against 4,520 mRNAs), each containing 5–7 different shRNA constructs against one mRNA. Lentivirus production, titre validation, infection, and selection of transduced cells in the presence of puromycin (1 µg/ml) were performed according to the manufacturer’s instructions. The multiplicity of infection (MOI) was adjusted to 0.2–0.3 to avoid multiple infections of a single cell. In each experiment, 6–7 × 10^6^ independent cell clones were obtained for each module. The cell clones were cultured for several days in the presence of puromycin until reaching 1.5–2 × 10^8^ cells, then split at a density of 2.5 × 10^4^ cells/cm^2^ in the presence of 1 mM db-cAMP without puromycin. After further culturing for 24 h, the cells were trypsinised and washed with ice-cold PBS. Then they were fixed in methanol-PBS (9:1, v/v) and kept on ice until the next step. Three biological replicates of lentiviral infection were performed.

### Cell processing for staining

The fixed cells were washed several times in ice-cold PBS with 10% FBS. PBS containing 0.1% Tween20, 1% normal goat serum and 10% FBS was used for blocking nonspecific binding of the antibodies used, as well as for washing the cells. Anti-collagen IV antibody (Abcam, ab19808) was diluted in the blocking buffer at a concentration of 10 µg/ml and incubated for 1 h on ice. After washing the cells, goat anti-rabbit IgG antibody conjugated with Alexa488 (Molecular Probes) was used for visualisation of the type IV collagen expression. The cells were washed with the blocking buffer and passed through Cell Strainer (BD Biosciences, 40 µm pore size). Each final stained preparation contained 2.0–2.2 × 10^8^ cells.

### Isolation of cells using FACS

Primarily gated cells by referring FSC-A and SSC-A were separated according to the fluorescence intensity of Alexa488 using a FACSAria II (BD Biosciences) flow cytometer and FACS Diva software (BD Biosciences). Approximately 10% of the high-intensity cells (2 × 10^7^ cells) and the corresponding input (2 × 10^7^ cells) were pooled for each replicate.

### HTS analyses of pooled fractions

Genomic DNA was extracted from the sorted cells using standard procedures. Shared DNA fragments containing bar-coded shRNA constructs were subjected to two-round PCR amplification using the primer pairs listed in Supplementary Table S8, according to the instructions provided by Cellecta. Libraries were prepared according to Illumina protocols, and 2 × 10^7^ reads were sequenced on Illumina HiSeq1000 using the primer listed in Supplementary Table S8. Sequence data were analysed using Barcode Deconvoluter software (Cellecta) and read counts for each shRNA construct were extracted.

### Data manipulation to extract the final result

The read count data from the input and the Alexa488-high-intensity fractions were further processed by quantile normalisation^33^. The mean log2-fold change (log2-fc) and P-value (Student’s *t*-test) for differences between the input and the Alexa488-high-intensity fractions were then calculated for each shRNA construct from three biological replicates. shRNA constructs demonstrating significant enrichment (log2-fc > 0.3 and P < 0.01) in the Alexa488-high-intensity fraction were extracted (1,276 genes; Supplementary Table S1). Data were then subjected to gene ontology analysis to select genes encoding nuclear or cytosolic proteins (PANTHER classification system, cellular component enrichment analysis^34,35^; Supplementary Table S2). When the selected data were compared with the F9 cell- and the ESC-expression datasets^24,36^, 434 genes with mean log2-counts per million (log2-cpm) values >3 (Supplementary Tables S3 and S4) were kept as the final candidate genes. These 434 genes were then reanalysed using the PANTHER classification system (Fig. 3A and Supplementary Table S5).

### Further validation by HTS analyses

From the final 434 candidate genes, *Prdm5* and *Setd5* were selected for further validation. For this, publically available naïve and primed ESC RNA-seq data^36^ were analysed using STAR^37^ and edgeR^38^. The publically available ChIP-seq data of histone modifications were mapped using Bowtie2^39^, and metagene analyses of the mapped ChIP-seq data^36^ were visualised by either Integrative Genomics Viewer (IGV)^40^ or ngs.plot^41^. All of the control ESC high-throughput sequence data were downloaded from GSE23943^36^. Differentially expressed genes between wild-type and *Prdm5*-knockout ESCs (GSE51553)^30^ were analysed using STAR, and edgeR (|log2-fc| > 1, P < 0.01; Supplementary Table S6). Differentially expressed genes between wild-type and *Setd5*-knockout ESCs were obtained from previously published data^31^ (|log2-fc| > 1, P < 0.01; Supplementary Table S7). The ESC modules^25^ and genes with bivalent chromatin were described previously^42^. Venn diagrams were drawn using Venny (http://bioinfogp.cnb.csic.es/tools/venny/) and statistical analysis of genes that were categorised in the PRC module against those in either the Myc module or the Core module was performed using the Chi-square test.

### *In silico* characterisation of reporter genes

ChIP-on-Promoter array data were used for isolation of PRC2-enriched genes in F9 cells (median value >0.4; Squazzo *et al*.^23^). Differentially expressed gene (DEG) data were reanalysed using STAR^37^ and edgeR^38^ (log2-fc > 4, log2-cpm > 2; GSE56893, Chatagnon *et al*.^24^) (Supplementary Table S4).

## Electronic supplementary material

Figures S1-S4

Tables S1-S8
