# Supplementary material for: Unbiased shRNA screening, using a combination of FACS and high-throughput sequencing, enables identification of novel modifiers of Polycomb silencing
Source: Sci Rep. 2018 Aug 14;8:12128. doi: 10.1038/s41598-018-30649-6 (PMC6092423; doi:10.1038/s41598-018-30649-6)
Supplement: Supplementary file 1 — Figures S1-S4 [file 41598_2018_30649_MOESM1_ESM.pdf]

Supplementary Information

Unbiased shRNA screening, using a combination of FACS and high-throughput sequencing, enables identification of novel modifiers of Polycomb silencing

Kenichi Nishioka, Hitomi Miyazaki, and Hidenobu Soejima

Figure_S1

A

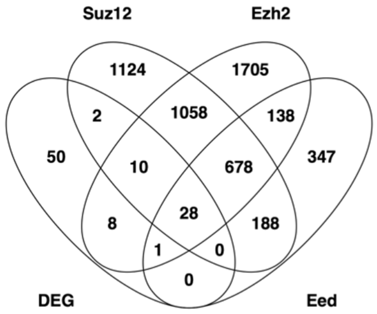

B

Gene	log2-fc_RA	Log2-cpm	P-value
Col4A1	3.76	9.45	5.148E-06
Col4A2	3.95	8.84	2.128E-06
Igf2	3.72	6.38	6.640E-06
Foxq1	6.97	6.30	2.347E-12
Cyp26A1	4.62	6.30	1.067E-07
Gata6	5.11	5.68	1.234E-08
Slc16A2	8.25	5.60	1.140E-14
Sfrp5	5.80	5.57	5.016E-10
Pdgfra	4.54	4.82	2.022E-07
Foxa1	5.45	4.62	4.240E-09
Zfp503	5.39	4.60	5.378E-09
Smarca2	3.03	4.41	1.443E-04
Sox17	5.34	4.19	8.491E-09
Hoxb2	8.20	4.09	2.150E-13
Hoxb9	3.09	3.93	1.222E-04
Hoxa5	6.57	3.81	8.804E-11
Nrp1	4.72	3.81	1.254E-07
Irs4	6.29	3.78	2.823E-10
Foxa2	5.79	3.61	2.426E-09
Runx1	5.11	3.48	3.526E-08
Foxf2	6.26	3.40	6.648E-10
Hoxb4	5.56	3.39	7.340E-09
Adra2B	3.51	3.32	2.633E-05
Cyp1B1	7.21	3.14	6.583E-11
Fgf10	4.78	3.14	1.890E-07
2510009E07Rik	3.17	3.09	1.074E-04
Gfra1	5.01	3.08	6.829E-08
Has2	3.88	3.06	5.365E-06

C

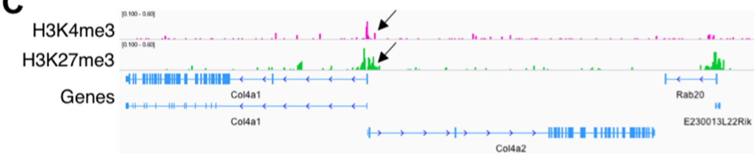

Figure S1. In silico characterisation of *Col4a1/a2* as the reporter gene. (**A**) Venn diagram comparing ChIP-on-Promoter array data (median value > 0.4; Squazzo et al.[23]) and DEG data (log2-fc > 4, log2-cpm > 2; Chatagnon et al.[24]) after 48 h of RA treatment (Supplementary Table S4). (**B**) All 28 of the positive genes that are plotted in Figure 1A. (**C**) Chromatin context of *Col4a1/a2* in mouse ESCs. Normalized HTS read coverage of histone H3K4me3 (magenta) and H3K27me3 (green) in *Col4a1/a2* loci are shown. Arrows indicate bivalency of the *Col4a1/a2* promoter region. *Col4a1/a2* and *Rab20* are members of the PRC module[25].

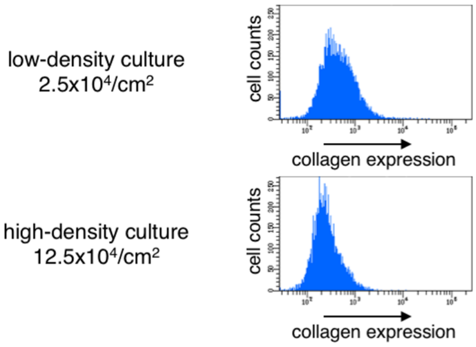

Figure S2. Type IV collagen induction level is dependent on culture conditions. Lentiviral transduced F9 cells at the indicated cell density were analysed by flow cytometry.

Figure_S3

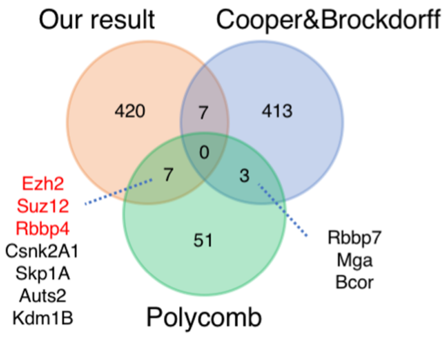

Figure S3. Comparison of similar screenings using representative Polycomb-target genes as the reporter. A Venn diagram representing the relationship between our results, using F9 cells and collagen IV expression as the marker of nuclear-protein-encoding genes, the screening results of Cooper and Brockdorff[17], using ESCs with *Gata6* promoter-driven neomycin-resistant gene expression as the marker, and Polycomb-group and related genes[2]. *Ezh2* and *Suz12*, and *Rbbp4*, major components of PRC2, are highlighted in red.

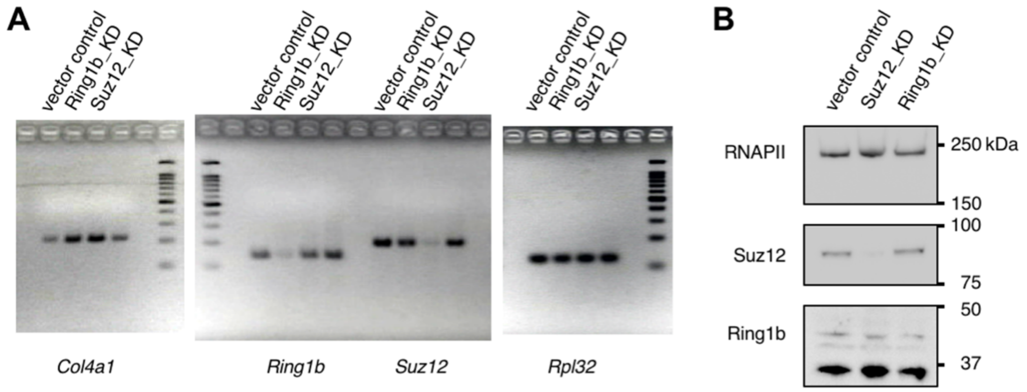

Figure S4. Raw data from conventional RT-PCR and Western blots. (**A**) Refer to Figure 1F. Analyses of expression of the indicated mRNAs in either *Suz12*- or *Ring1b*-knockdown (KD) F9 cells. (**B**) Refer to Figure 1G. Almost-full data for each strip are shown.
